# Longitudinal DNA methylation dynamics as a practical indicator in clinical epigenetics

**DOI:** 10.1186/s13148-021-01202-6

**Published:** 2021-12-13

**Authors:** Shohei Komaki, Hideki Ohmomo, Tsuyoshi Hachiya, Yoichi Sutoh, Kanako Ono, Ryohei Furukawa, So Umekage, Yayoi Otsuka-Yamasaki, Kozo Tanno, Makoto Sasaki, Atsushi Shimizu

**Affiliations:** 1 Division of Biomedical Information Analysis, Iwate Tohoku Medical Megabank Organization, Disaster Reconstruction Center, Iwate Medical University, 1-1-1, Idaidori, Yahaba, Iwate 028-3694 Japan; 2 Department of Biology, Research and Education Center for Natural Sciences, Keio University, 4-1-1 Hiyoshi, Kohoku-ku, Yokohama, Kanagawa 223-8521 Japan; 3 Division of Clinical Research and Epidemiology, Iwate Tohoku Medical Megabank Organization, Iwate Medical University, Iwate, Japan; 4 Department of Hygiene and Preventive Medicine, Iwate Medical University, 1-1-1 Idaidori, Yahaba, Shiwa, Iwate 028-3694 Japan; 5 Iwate Tohoku Medical Megabank Organization, Iwate Medical University, Iwate, Japan; 6 Division of Ultrahigh Field MRI, Institute for Biomedical Sciences, Iwate Medical University, 1-1-1 Idaidori, Yahaba, Shiwa, Iwate 028-3694 Japan; 7 Division of Biomedical Information Analysis, Institute for Biomedical Sciences, Iwate Medical University, 1-1-1 Idaidori, Yahaba, Shiwa, Iwate 028-3694 Japan

**Keywords:** Blood DNA methylation, EWAS marker likelihood, Illumina 450 K beadchip, Temporal stability

## Abstract

**Background:**

One of the fundamental assumptions of DNA methylation in clinical epigenetics is that DNA methylation status can change over time with or without interplay with environmental and clinical conditions. However, little is known about how DNA methylation status changes over time under ordinary environmental and clinical conditions. In this study, we revisited the high frequency longitudinal DNA methylation data of two Japanese males (24 time-points within three months) and characterized the longitudinal dynamics.

**Results:**

The results showed that the majority of CpGs on Illumina HumanMethylation450 BeadChip probe set were longitudinally stable over the time period of three months. Focusing on dynamic and stable CpGs extracted from datasets, dynamic CpGs were more likely to be reported as epigenome-wide association study (EWAS) markers of various traits, especially those of immune- and inflammatory-related traits; meanwhile, the stable CpGs were enriched in metabolism-related genes and were less likely to be EWAS markers, indicating that the stable CpGs are stable both in the short-term within individuals and under various environmental and clinical conditions.

**Conclusions:**

This study indicates that CpGs with different stabilities are involved in different functions and traits, and thus, they are potential indicators that can be applied for clinical epigenetic studies to outline underlying mechanisms.

**Supplementary Information:**

The online version contains supplementary material available at 10.1186/s13148-021-01202-6.

## Background

DNA methylation is the most widely studied form of epigenetic modification. The DNA methylation status of cytosine phosphate guanine (CpG) dinucleotides especially has been attracting attention in clinical epigenetic studies [1]. DNA methylation of CpG modulates transcriptional regulation, including gene silencing and alternative splicing, and plays a role in genome stability [1]. DNA methylation status can change in response to external factors, such as lifestyle and stress, and is involved in disease onset and development. Owing to the development of cost-effective techniques for determination of genome-wide DNA methylation, such as Illumina HumanMethylation450 BeadChip (HM450k) and Illumina MethylationEPIC (EPIC) microarray kits, large-scale epigenome-wide association studies (EWAS) have been conducted to explore the underlying epigenetic mechanisms of a variety of diseases, environmental stresses, and other measurable traits. As a result, numerous CpGs have been identified as epigenetic markers of various traits [2]. More recently, DNA methylation status has been used to evaluate the biological age (epigenetic age) of individuals [3], and a reversal of epigenetic aging could be induced by artificial stimuli [4].

One of the fundamental assumptions in these epigenetic studies is that DNA methylation varies within each individual with time. Thus, in order to properly interpret the clinical implications of the observed DNA methylation change—for example, before and after disease onset, stress exposure, and therapeutic intervention—we need to understand whether and how DNA methylation usually changes under ordinary environmental and clinical conditions. A small number of studies have reported on how many and which regions of the genome are susceptible to changes in DNA methylation with time using HM450k and EPIC: CpGs in the human genome are largely stable longitudinally [5, 6], CpGs with intermediate DNA methylation level or present within CpG-poor regions are likely to be stable [7], and the temporal changes in the DNA methylation status are largely caused by a change in the type of cell components [8]. Due to poor reproducibility, it has been proposed that highly variable CpGs that vary temporally within an individual, relative to those that vary between individuals, should be excluded prior to analyses [5]. While these studies provided significant insights into the stability of DNA methylation, which can contribute to the EWAS design and its interpretation, these studies largely observed only a few specific genes or only compared two time points several months apart. Moreover, no studies have delved into unstable CpGs that, potentially, have significant biological roles, but were treated as noise on statistical analyses. Thus, a more comprehensive study based on densely measured DNA methylation data is needed to understand the global nature of DNA methylation dynamics.

The study by Furukawa et al. [9], which, to our knowledge, provides the densest longitudinal data to date, analyzed the temporal DNA methylation and gene expression changes obtained from blood samples collected 24 times over three months from two different men. They evaluated the contribution of DNA methylation to gene expression dynamics (expression quantitative trait methylation; eQTM) and suggested that DNA methylation is stable and barely explains the gene expression dynamics.

In this study, we reanalyzed the longitudinal DNA methylation data obtained from Furukawa et al. and characterized the overall dynamics of DNA methylation—not only eQTM—at a finer-scale. We aimed to provide an insight into the dynamics and interpretations of DNA methylation that are applicable to clinical epigenetic analysis.

## Materials and methods

### DNA methylation data

The DNA methylation data was obtained from Furukawa et al. [9]. Briefly, blood samples were collected 24 times over a period of 84 d from two apparently healthy men. The PBMCs and monocytes were extracted, their genomic DNA was isolated, and the DNA methylation levels of genome-wide CpGs were measured using HM450k. Thus, 96 datasets were generated (2 individuals × 2 sample types × 24 time points). Samples were not randomly loaded but were ordered by the blood collection date on the microarrays. Probe-type bias in each dataset was corrected using beta-mixture quantile (BMIQ) normalization method that was implemented in the R package “wateRmelon” [10]. DNA methylation levels were then converted into percentages. Furukawa et.al. further estimated blood cell-type composition (CD4^+^ and CD8^+^ T cells, natural killer cells, monocytes, granulocytes, and B-cells, Additional file 1: Fig. S1); this data was also used in the current study. Blood samples were also subjected to serological tests, the results of which are summarized in Additional file 2: Table S1.

**Fig. 1:**
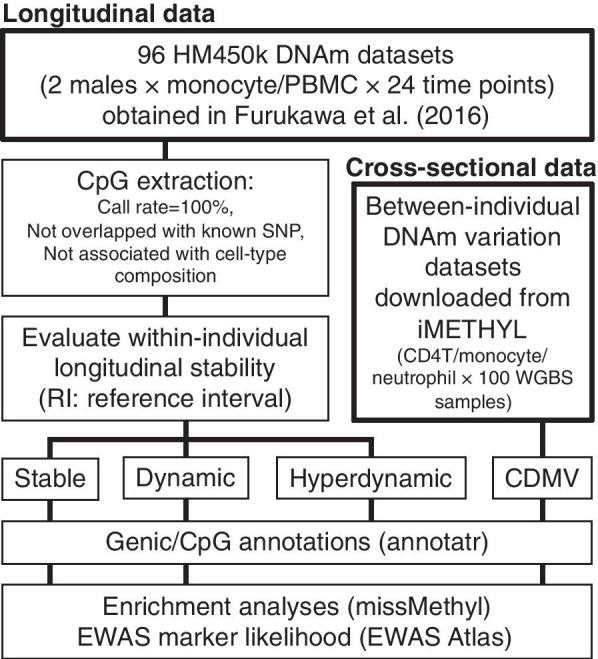
Study design. There are two sources of datasets analyzed in this study: longitudinal datasets from Furukawa et al. [9] and cross-sectional datasets from the iMETHYL database

In the current study, CpGs that had missing data on DNA methylation in any of the 96 datasets or those overlapping with SNPs found in Japanese populations with MAF ≥ 5% were excluded [11] (Fig. 1). Furthermore, the apparent DNA methylation level can be influenced by changes in cell-type composition [12]. Thus, to accurately evaluate the stability/dynamics of the DNA methylation status of CpG sites, CpGs showing DNA methylation changes synchronized with cell-type component changes were excluded. To identify CpGs associated with cell-type components, analysis of variance (ANOVA) was performed as in Furukawa et al. [9] whereby the fits of two regression models were compared. The two models compared were as follows: (1) a simple linear regression with DNA methylation level as a dependent variable and a fixed value of 1 as an explanatory variable; (2) a multiple linear regression with the estimated proportion of each six cell types specified as explanatory variables. If the multiple regression model fit was significantly better (*p* value < 0.05), the CpG was considered to be associated with the cell-type composition.

### Fluctuation evaluation

To evaluate the magnitude of change in the differential longitudinal DNA methylation between individuals (samples A and B) and sample types (PBMC and monocytes), we first performed principal component analysis (PCA) using all 96 datasets.

As an indicator of longitudinal DNA methylation change, we applied reference intervals (RI) that were originally proposed to evaluate the difference in DNA methylation between individuals [13]. RI is defined as the difference between the 95th and 5th percentiles of DNA methylation levels across individuals (between-individual RI, Additional file 1: Fig. S2) [13]. In this study, within-individual RI was calculated as the difference between the 95th and 5th percentiles across 24 time points in the same individual (Additional file 1: Fig. S2). Intraclass correlation coefficients have been used to evaluate the DNA methylation dynamics within individuals [5, 7, 8, 14]; however, it is calculated based on both within- and between-individual variation, and thus, is not applicable to the datasets used in this study, which consist of data from only two individuals.

To characterize the pattern of the longitudinal DNA methylation change, within-individual RI and mean DNA methylation level of each CpG were compared across 24 time points. In addition, since variation in within-individual DNA methylation can contribute to an apparent variation in between-individual methylation patterns, the within- and between-individual RIs were further compared. Between-individual RI, which was calculated based on whole genome bisulfite sequencing of ~ 100 Japanese individuals, was downloaded from the iMETHYL database [15]. Since between-individual RI for PBMC was unavailable in iMETHYL, only the within- and between-individual RIs for monocytes were compared. Furthermore, only CpGs that overlapped between the present datasets and that from iMETHYL were considered.

For the downstream analyses, we extracted stable, dynamic, and hyperdynamic CpGs from each of the four samples (2 individuals × 2 sample types). CpGs that exhibited within-individual RI < 1% and 10–50%, which are approximately the 1st and 99th percentiles across all CpGs, are defined as stable and dynamic CpGs, respectively. CpGs with RI ≥ 50% were defined as hyperdynamic CpGs.

To consider between-individual RI in addition to within-individual RI in the downstream analyses, between-individual RIs for CD4^+^ T lymphocytes (CD4T) and neutrophils as well as monocytes were considered from the iMETHYL database. As suggested by Hachiya et al. [13], CpGs with between-individual RI > 30% were regarded as between-individual variable (common DNA methylation variation; CDMV) CpGs.

### CpG site annotations and enrichment analyses

The CpG and genic annotations for each CpG category were performed using the R package “annotatr” [16]. Because some CpGs have multiple neighboring genes, a single CpG may be assigned multiple genic annotations. To account for this possible bias, fractional counting was employed, where each genic annotation count was divided by the total number of annotations of the given CpGs: i.e., if a CpG is mapped to a promoter region of gene A and an exon of gene B, annotation counts will be 1/2 and 1/2.

Furthermore, enrichment analyses were performed since the CpGs present in the genic or upstream regions can especially affect the transcriptional pattern of neighboring genes [17]. Genes with stable, dynamic, and CDMV CpGs in their genic and upstream regions were considered separately. In total, 11 enrichment analyses were performed that included those for stable and dynamic CpGs in each of the four samples (2 individuals × 2 sample types), and CDMV CpGs in each of the additional three cell types (CD4T, monocytes, and neutrophils). In the analyses, four frameworks, including Gene Ontology (GO) annotations [18, 19] of biological process (BP), cellular component (CC), and molecular function (MF) and Kyoto Encyclopedia of Genes and Genomes (KEGG) pathways [20] were applied. The analyses were carried out using the gometh() function in the R package “missMethyl” [21]. This function can control the bias arising from multiple genes being annotated on a single CpG or from the difference in the number of probes designed for each gene. In the analyses, only CpGs located in gene bodies and upstream regions were considered. Terms and pathways with a false discovery rate (FDR)-adjusted *p* value < 0.05 from the Wallenius’ noncentral hypergeometric test were considered to be significantly enriched. Enrichment analysis for hyperdynamic CpGs was not performed because of their limited number. Instead, we focused on two genes annotated for four CpGs, which were hyperdynamic across all four samples.

In the HM450k, two types of assays were employed: Infinium I and II. Infinium I assay uses two types of probes, whereas Infinium II uses a single type of probes per CpG locus. To characterize the differences in DNA methylation stability attributed to the microarray design, the ratio of both Infinium I and II assays in each CpG category was estimated. In addition, GC contents of microarray probes were calculated in each CpG category.

### Overlap evaluation with EWAS markers

To infer the relationships between the stability of DNA methylation and various traits, including those for diseases and environmental exposures, the overlap between CpGs in each category (stable, dynamic, hyperdynamic, and CDMV) or annotation (CpG island, shore, shelf, and open sea) and EWAS markers was evaluated. EWAS marker list was downloaded from EWAS Atlas [2] on July 1st, 2020. The EWAS marker likelihood for each CpG category was calculated by dividing the number of CpGs overlapped with EWAS marker by the number of non-overlapped CpGs in the category. By comparing the likelihoods of all HM450k probes (background) using Fisher’s exact test, we estimated the odds ratio of each CpG category.

In the EWAS Atlas dataset, various kinds of traits were listed, which must have different underlying epigenetic mechanisms and different causes for DNA methylation change. Therefore, EWAS marker likelihood for each CpG category was further evaluated by focusing on each EWAS trait separately. The EWAS traits with five or more published studies and 100 or more identified CpG markers were selected and the EWAS marker likelihood was calculated with the same method as that used for the entire EWAS Atlas dataset. The EWAS Atlas is based on manually curated data [2]; however, we cannot rule out the presence of studies without controlling confounders in the dataset that could distort the EWAS marker likelihood. To reduce the possible bias, trait-level meta-analyses were performed. First, the odds ratio was calculated for each study of each trait, and then random-effect meta-analyses were conducted for odds ratios per trait using the R package “metafor” [22] with DerSimonian Laird estimator. The between-study heterogeneity was assessed by Cochran’s Q test using “metafor” with a significant threshold P value of 0.05. As the number of hyperdynamic CpGs was small and not applicable to the trait-specific analysis, the category was excluded from the analyses.

## Results

### Characteristics of DNA methylation fluctuation

**Fig. 2:**
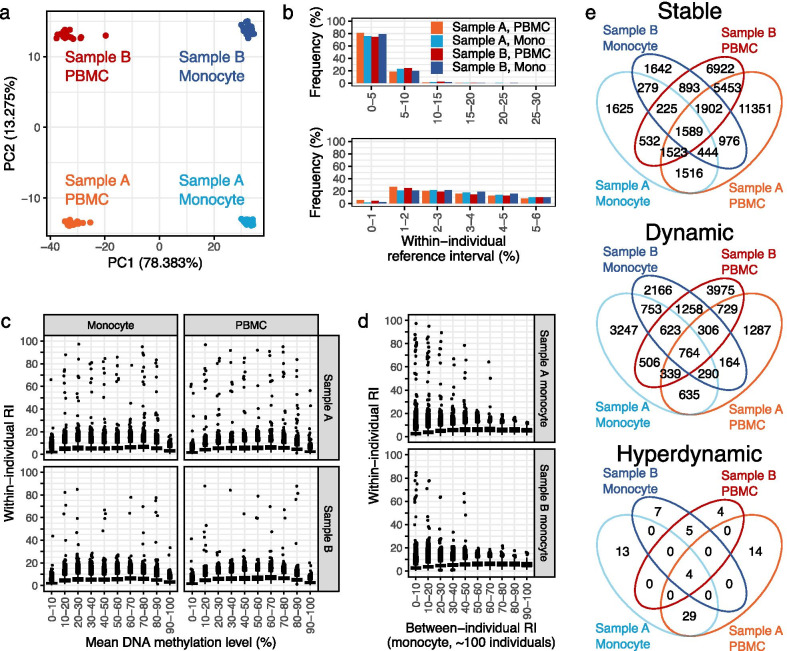
Global longitudinal DNA methylation change in four samples. **a** PCA plot based on 96 DNA methylation datasets. **b** Distribution of within-individual reference interval (RI) of each sample. Data for RI ≥ 30 is not represented as the bars were not visible at the present scale. **c** Box plots showing the relationship between mean and variation of DNA methylation level within three months. **d** Box plots showing the relationship of between- and within-individual reference intervals in monocytes. Between-individual RI was downloaded from the iMETHYL database which was calculated based on ~ 100 individuals. In each box plot, outliers were depicted as points. **e** Number of CpG in each category and overlaps between them

Originally, the longitudinal dataset obtained from two males contained 484,376 CpGs from which 791 with missing values and 16,058 associated with cell-type components were removed. From the resultant 467,527 CpGs, 436,147 CpGs that overlapped with iMETHYL WGBS data but did not overlap with known SNPs were extracted and analyzed in this study. PCA showed that global DNA methylation patterns of the same individual and the same sample type were similar to each other (Fig. 2a). Indeed, ~ 80% of all CpGs exhibited within-individual RI < 5%, approximately 20% of CpGs exhibited RI 5–10%, and a small fraction of CpGs exhibited larger within-individual RI (Fig. 2b and Additional file 1: Fig. S3). Majority of CpGs analyzed in this study exhibited DNA methylation levels close to 0 or 100% (Additional file 1: Fig. S3).

CpGs with intermediate mean DNA methylation levels tended to exhibit larger within-individual RI rather than those with extreme levels that approximated to either 0 or 100% (Fig. 2c). In monocytes, furthermore, CpGs with intermediate between-individual RI tended to exhibit larger within-individual RI (Fig. 2d). The trends that were consistent across samples, however, differ from a previous study [5]. This difference may have resulted from the fact that the previous study has normalized DNA methylation levels and adjusted the longitudinal variation for between-individual variation. CpGs with extreme within-individual RI (RI > 50%) tended to exhibit lower between-individual RI (Fig. 2d).

**Fig. 3:**
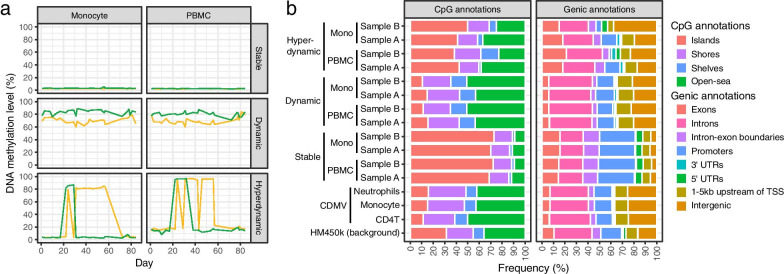
Characteristics of CpG categories. **a** longitudinal DNA methylation changes of three randomly-selected CpGs of stable, dynamic, and hyperdynamic categories in two individuals (green and yellow). Note that some stable CpGs exhibited stable DNA methylation level close to 100%. **b** CpG and genic annotation compositions in each category and sample. CDMV: common DNA methylation variation (between-individual variable CpG)

The number of CpGs assigned to stable, dynamic, and hyperdynamic categories were 24,754/7,733/19,039/7,950 (PBMC (sample A)/monocyte (sample A)/PBMC (sample B)/monocyte (sample B)), 4,514/7,157/8,500/6,324, and 47/46/13/16, respectively (Fig. 2e and Additional file 2: Table S2). Stable, dynamic, and hyperdynamic CpGs showed distinct patterns of longitudinal DNA methylation changes (Fig. 3a). As expected from their definitions, stable CpGs had a stable DNA methylation status, and dynamic CpGs exhibited considerable DNA methylation fluctuation that approximately ranged within 20%. Hyperdynamic CpGs showed a binary state of DNA methylation close to either 0 or 100%. The patterns of longitudinal DNA methylation change in dynamic and hyperdynamic CpGs were synchronized between PBMCs and monocytes within individuals.

Among dynamic CpGs identified in any four samples, 26–51% overlapped with between-individual variable (CDMV) CpGs, whereas only 3–7% of CDMV CpGs overlapped with dynamic CpGs (Additional file 1: Fig. S4). Hyperdynamic CpGs also exhibited notable overlap with CDMV CpGs (12–26%). Nearly no stable CpGs were found to overlap with CDMV CpGs.

### Annotations and enriched functions

CpG annotations showed that CDMV CpGs were poor in CpG islands and abundant in open sea, while stable CpGs were abundant in CpG islands and poor in shelves and open sea. Meanwhile, dynamic CpGs were abundant in open sea and poor in islands, and hyperdynamic CpGs were abundant in islands, compared to the entire CpGs covered by HM450k (Fig. 3b). These trends were consistent across four samples or three cell-types (Fig. 3b and Additional file 1: Fig. S5) and are largely consistent with previous studies although the index of variation is different [5, 8].

The genic annotation component was also consistent across samples or cell types: CDMV CpGs were abundant in intergenic regions and poor in exon and promoter regions; stable CpGs were abundant in exons, intron–exon boundaries, promoters, and 5’UTRs and poor in introns, 1 to 5 kb-upstream regions of TSS, and intergenic regions; dynamic CpGs were abundant in introns, 1–5 kb upstream of genes, and intergenic regions and poor in exon, promoter, and 5’ UTR regions; and hyperdynamic CpGs were abundant in exon and intergenic regions (Fig. 3b).

Enrichment analyses showed that metabolism-related functions were overrepresented among genes with stable CpGs, whereas only six GO:BP and GO:CC terms that were not common across samples were overrepresented among genes with dynamic CpGs (Additional file 1: Figs. S6–S9 and Additional file 2: Tables S3–S6). Except for dynamic CpGs, enriched terms and pathways for each CpG category were largely consistent across three cell types and four samples (Additional file 1: Fig. S10).

Among the four hyperdynamic CpGs that were common across the four samples, two CpGs were in intergenic regions and the other two (cg07376282 and cg22588144) were located in an exon of *GPR37* and an upstream region of *ISM1*, respectively. *ISM1* is an angiogenesis inhibitor, and two GO terms were associated with the gene (extracellular region and negative regulation of angiogenesis). *GPR37* is a G protein-coupled transmembrane receptor with suggested roles in the brain and is related to the pathogenesis of neurological disorders, including Parkinson’s disease and autism spectrum disorders [23–25]. Twenty-nine GO terms were associated with this gene (e.g., ubiquitin protein ligase binding, G protein-coupled receptor activity, neuropeptide signaling pathway, and heat shock protein binding) (Additional file 2: Table S7).

Characteristics of probes were different among CpG categories (Additional file 1: Fig. S11). The stable CpG category exhibited a higher ratio for Infinium I assay and a significantly greater GC content compared to the HM450k background. Enrichment analyses separately performed for Infinium I and II CpGs in the stable category showed that the enriched terms and pathways in Infinium I CpGs mostly overlapped with those in Inifium II CpGs (Additional file 1: Fig. S11). The dynamic CpG category exhibited a comparable assay ratio and a significantly smaller GC content. The hyperdynamic CpG category exhibited a higher assay ratio, but no significant difference in GC content was observed except for a single sample (Additional file 1: Fig. S11).

### EWAS markers and DNAm fluctuations

**Fig. 4:**
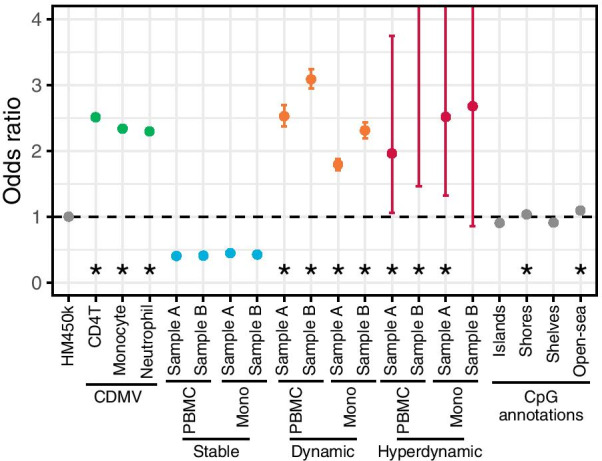
EWAS marker likelihood for CpGs in each CpG category. For the purpose of visibility, upper limit of y axis was set to 4. Untrimmed plot is presented in Additional file 1: Fig. S12. Asterisks were given for categories with positive odds ratio which is significantly deviated from 1. CDMV: common DNA methylation variation (between-individual variable CpG)

When all EWAS markers included in the EWAS Atlas dataset were considered, CpGs in CpG shores and open-sea exhibited slightly higher likelihoods (ORs = 1.035 (shore), 1.095 (open-sea)) rather than those of islands and shelves (ORs = 0.905 (island), 0.910 (shelf)) (Fig. 4 and Additional file 1: Fig. S12). However, a much larger magnitude of likelihood was shown in dynamic and CDMV CpGs. Odds ratios of CDMV CpGs were almost consistent across three blood cell types, meanwhile, those of dynamic CpGs were relatively different among four samples. The number of hyperdynamic CpG was small and that resulted in broader confidence intervals; however, odds ratios of hyperdynamic CpGs tended to be higher than 1. Meanwhile, the odds ratios of stable CpGs were lower than 1 across all four samples.

**Fig. 5:**
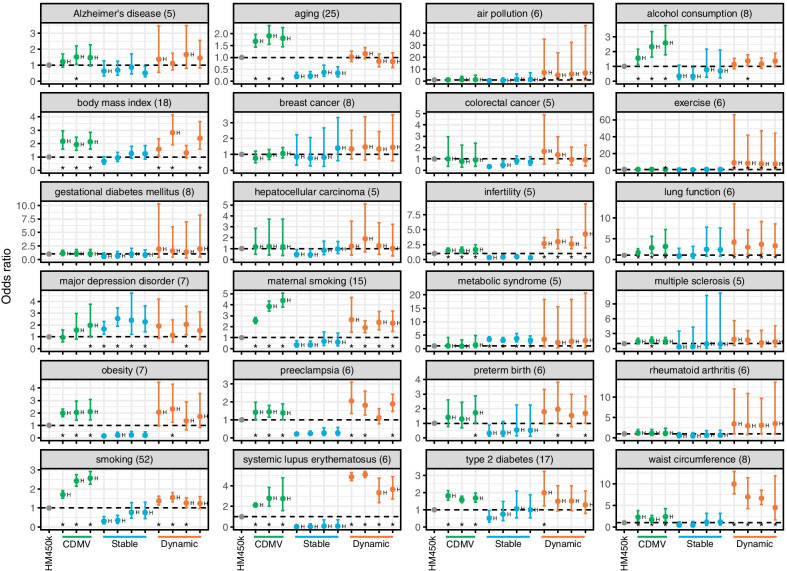
Likelihoods of trait-specific EWAS markers for each CpG category resulted from meta-analyses. Gray, blue, and orange plots represent odds ratios for overall-HM450k, CDMV, stable, and dynamic CpGs, respectively. Plot order is the same as that of Fig. 4. Asterisks were given for categories with positive odds ratio which is significantly deviated from 1. H indicates the presence of between-study heterogeneity of odds ratio. CDMV: common DNA methylation variation (between-individual variable CpG). Numbers in parentheses are numbers of studies considered here

EWAS trait-specific analyses also showed that dynamic and CDMV CpGs generally exhibit higher marker likelihoods while stable CpGs exhibit lower likelihoods (Additional file 1: Fig. S13). EWAS trait-specific meta-analyses showed similar patterns of likelihoods (Fig. 5). The meta-analyses indicated the presence of between-study heterogeneity of odds ratio in a majority of traits. Considering the results consistent across the three cell types or four samples, likelihoods of CDMV were significantly high in traits related to aging, alcohol consumption, BMI, obesity, preeclampsia, smoking, and type 2 diabetes, while likelihoods of stable CpGs were high in traits associated with major depression disorder and metabolic syndrome. Additionally, likelihoods of dynamic CpGs were high in traits related to air pollution and exercise. Both CDMV and dynamic CpG categories exhibited significantly high likelihoods for traits related to infertility, lung function, maternal smoking, systemic lupus erythematosus, and waist circumference.

All four hyperdynamic CpGs common across the four samples (cg04955116, cg07376282, cg22588144, cg27326062) were reported as EWAS markers of air pollution (PM 2.5) [26], rheumatoid arthritis [27], infertility (studies on sperm) [28–31], exercise [32], and Kabuki syndrome [33]. A single hyperdynamic CpG in *GPR37* (cg07376282) was reported as an epigenetic marker for infertility. It is notable that this dynamic CpG was reported as the marker in four studies [28–31]. However, it is unclear whether the hyperdynamic DNA methylation change observed in blood also occurs in testes beyond the blood-testis barrier.

## Discussion

### Dynamics of DNA methylation profile

In PCA analysis, the 96 blood DNA methylation datasets were clearly segregated into four groups corresponding to the four samples (2 individuals × 2 sample types), demonstrating a minor longitudinal change in the global DNA methylation profile across 24 time-points. Supportive evidence was obtained from the distribution of within-individual RI; the majority (~ 80%) of CpGs in HM450k were relatively stable, exhibiting within-individual RI < 5%. These results are consistent with previous studies [5, 6]. However, according to the EWAS Atlas database, ~ 10% of EWAS markers showed < 5% of DNA methylation difference between the case and control groups. Our results suggest that the degree of differential DNA methylation observed between groups in previous studies can occur within the individual during several months, and thus, we should be more cautious about the higher possibility of false positives, especially in longitudinal studies.

### Characteristics of CpGs with different stabilities

Assignments of CpGs into stable, dynamic, and hyperdynamic categories were similar between the four samples. In addition, based on CpG and genic annotations, the annotation components of each category were also similar between the four samples. Moreover, the patterns of change in longitudinal DNA methylation were synchronous between PBMCs and monocytes within individuals. These results strongly indicate that the observed DNA methylation dynamics are not from an experimental error, but rather, arise from biological consequences.

Since stable CpGs are relatively abundant in the genic and upstream regions, they probably ensure stable gene transcription. Dynamic CpGs are abundant in introns and non-genic regions. It is unclear whether and what functions the dynamic CpGs in these regions have in relation to transcription regulation. However, although a minor fraction, dynamic CpGs were also found in genic and upstream regions, and these are more likely to have functions in transcriptional regulation. Among genes with stable CpGs in their genic and promoter regions, gene ontology terms related to metabolic processes were highly enriched. Metabolism is a life-sustaining process which occurs in all living organisms, and thus, its regulation should not be disrupted in order to maintain a healthy condition [34]. Therefore, it is reasonable that the DNA methylation status of metabolism-related genes is stable.

It should be noted that differences in probe design may also have contributed to the observed longitudinal dynamics. The Infinium type II assay uses a single bead to measure DNA methylation status of CpGs and is more susceptible to technical noise than the Infinium type I assay that uses two beads. With the greater noise, type II CpGs may have a lower probability of meeting the definition of a stable CpG (RI < 1%). In addition, it has been reported that noise reduces the dynamic range of DNA methylation levels [35], and thus, hyperdynamic change (RI ≥ 50%) in type II CpGs may have been less detectable. However, because results of enrichment analyses independently performed for types I and II largely overlapped, the major findings of this study appear to be less biased by the differences in the probe. The higher GC contents in the stable CpG category and the lower GC content in the dynamic CpG category may be related to the larger and smaller proportions of CpG islands, respectively.

### Relationships with traits

As they are associated with specific GO terms and pathways, CpGs with different stability should also be correlated with different traits. Our study indicates that CpGs with different stability exhibit different degrees of EWAS marker likelihoods; stable CpGs exhibited lower likelihoods while dynamic and hyperdynamic CpGs exhibited higher likelihoods. Moreover, the higher likelihood observed for CDMV CpGs is consistent with a previous study [13].

#### Stable CpGs

Low likelihoods observed for stable CpGs indicate that the DNA methylation status of a stable CpG is not only longitudinally stable within various cells of the individual but is also relatively unchanged between groups of individuals with different conditions, such as between patients and healthy groups and environmentally stressed and not-stressed groups. A low likelihood of age-EWAS marker in stable CpGs further indicates that CpGs stable for three months might also be stable for a longer period. These stable CpGs were overrepresented in the genic and upstream regions. Moreover, metabolism-related functions were enriched among the genes with stable CpGs. Thus, stable CpGs probably ensure the stability of metabolism, which could be the fundamental basis of living organisms. Conversely, disruption of these CpGs, which should be stable both longitudinally and cross-sectionally, may be linked to major physical or mental disorders, and this was supported by trait-specific EWAS marker likelihood analyses of stable CpGs.

The likelihoods of EWAS markers for major depression disorder and metabolic syndrome were significantly high in stable CpGs without significant heterogeneity between studies. Since the co-occurrence of major depression disorder and metabolic syndrome (or its risk factors) is well recognized [36, 37], it should be carefully considered that epigenetic characteristics of patients with major depression disorder can indirectly represent those of with the onset of metabolic syndrome. There are four possible explanations proposed for the comorbidity [38–41]; first, the unhealthy lifestyle of psychiatric patients, including habitual smoking, alcohol consumption, and irregularities in diet, sleep, and physical activity may increase their risk of metabolic syndrome; second, medical treatments for patients with major depression disorder, such as antidepressants, are known to cause metabolic dysregulation; third, there are shared pathophysiological features in immunometabolic and endocrine systems have been reported that interplay with both psychiatric disorders and metabolic syndrome; fourth, shared genetic architectures between psychiatric disorders and cardio-metabolic traits have been reported based on genome-wide association studies, twin genetic studies, and polygenic risk-score analyses. In terms of DNA methylation, meanwhile, there were no stable CpGs common between the EWAS markers of major depression disorder (69 stable CpGs) and metabolic syndrome (102 stable CpGs). This result indicates that although a similar pattern of EWAS marker likelihood was observed for both metabolic syndrome and major depression disorder, there is no similarity between both the disorders at the CpG level, making it likely that the underlying epigenetic mechanisms for both are independent from each other.

A high likelihood of the metabolic syndrome EWAS markers for stable CpGs is consistent with the fact that the metabolism-related functions were enriched among genes with stable CpGs. It is highly possible that DNA methylation changes in these CpGs, which are supposed to be stable over time, are linked with metabolic disorders. However, other traits connected to metabolic abnormalities such as BMI, obesity, type 2 diabetes, and waist circumference, exhibited different patterns of likelihood in which CDMV and dynamic CpGs were significantly high. The difference of complexity and severity of these traits compared to metabolic syndrome may partially explain the observed difference in EWAS marker likelihoods. Metabolic syndrome is a complex disorder comprising of cardiovascular disease risk factors [42–44]. There is no single cause for the syndrome, but several related or unrelated factors cause metabolic syndrome. For diagnosis of metabolic syndrome, although several criteria have been proposed, multiple conditions are generally considered, including insulin resistance, obesity (e.g., waist circumference and body mass index), lipid abnormalities (e.g., high triglyceride levels and low high-density lipoprotein cholesterol levels), high blood pressure, and impaired fasting blood glucose [42–44]. Thus, metabolic syndrome is a more complex condition and is a more severe risk factor for cardiovascular diseases and type 2 diabetes [42–44]. Physical conditions that meet the criteria for metabolic syndrome, which implies that there are more serious risks, may be linked to DNA methylation disruption enriched in metabolism-related genes that are supposed to be stable in healthy individuals. The EWAS marker likelihood for CDMV was high in type 2 diabetes, which we cannot explain conclusively based on the current evidence. Whether the same trend is observed in diabetic patients with metabolic syndrome, and how stable CpGs are disrupted during the development of metabolic syndrome via obesity, may be an important stepping stone to understanding the underlying epigenetic mechanisms of metabolic disorders. Waist circumference and BMI are not clinical conditions but are measurements, which can vary among healthy individuals. This may explain the higher observed likelihoods in CDMV.

#### Dynamic CpGs

Dynamic CpGs showed high likelihoods of being EWAS markers of seven traits. Some of these traits can be linked to inflammatory and immune responses. For example, respiratory infection and air pollution are major factors affecting lung function [45, 46], air pollution has inflammatory effects [47], systemic lupus erythematosus is an autoimmune disease [48], and exercise has anti-inflammatory effects [49]. Although it was not statistically significant, rheumatoid arthritis, which is an autoimmune disease, also showed a similar pattern of likelihoods. Because of this consistency across multiple immune- and inflammatory-related EWAS traits, it is expected that dynamic CpGs may be associated with immune and inflammatory responses. Immune and inflammatory responses can occur in healthy individuals owing to various environmental stimuli, which is probably reflected by longitudinal DNA methylation changes in dynamic CpGs.

Being reported as EWAS markers indicates that these CpGs exhibit different DNA methylation status between exposed/patient groups and control groups, despite the nature of the instability even within healthy individuals. This indicates that DNA methylation of these CpGs, which are supposed to be dynamic in healthy individuals, are fixed at specific levels in individuals with autoimmune diseases or exposure, implying an epigenetic state of chronic immune activation or inflammation.

This study indicated the biological importance of dynamic CpGs as it showed certain relationships with physiological traits. However, as mentioned in a previous study [5], unstable DNA methylation status within individuals can be considered as noise in statistical analyses. For example, exercise EWASs listed in the EWAS Atlas dataset measured short-term epigenetic changes before and after interventions (intervention study period ranged from minutes to 6 months) [32, 50–53]. The CpGs which were reported to exhibit short-term DNA methylation change after the intervention can also dynamically change in a short period of time without intervention. Although these were not intervention studies, air pollution EWASs listed in the database also mainly focused on a short-term measure of air pollution (e.g., for 24 h [54]). In such studies focusing on short-term DNA methylation changes, the possibility of a mixture of true epigenetic markers and false findings should be carefully noted. In the present study, broader odds ratios of dynamic CpGs and significant between-study heterogeneity in these traits were observed among all four samples, also implying the higher possibility of false findings.

#### Hyperdynamic CpGs

Hyperdynamic CpGs were scarce in the human genome, and thus, their statistically robust characterization could not be achieved. However, the longitudinal pattern of DNA methylation change characterized by obvious switching-on and -off in epigenetic status implies that these hyperdynamic CpGs mediate or reflect systematic molecular regulations. For instance, a single hyperdynamic CpG was identified in *ISM1*, which encodes an angiogenesis inhibitor. Vascular system has a fundamental role in the delivery of gases and nutrients, and excessive or insufficient angiogenesis can cause a considerable problem [55]. Thus, angiogenesis must be strictly controlled to ensure physiological homeostasis, and indeed, is regulated by a dynamic balance between activators and inhibitors [56]. The epigenetic dynamics of *ISM1* upstream region detected by HM450k probes may play a part of the dynamic regulation.

Other three hyperdynamic CpGs common across four samples, as well as that in *ISM1*, were reported as EWAS markers of several traits of air-pollution (PM 2.5) [26], rheumatoid arthritis [27], infertility (studies on sperm) [28, 29, 31], exercise [32], and Kabuki syndrome [33]. Except for infertility, these traits can be closely linked to immune or (anti-)inflammatory functions, and these CpGs are potentially highly sensitive markers that provide a detailed reflection of immune or inflammatory responses. These potentials could be assessed by comparing DNA methylation status with immune and inflammatory status at the time of blood sampling.

## Limitations and implications for future studies

First, we note that this study was based on data from only two Japanese males, and thus, it remains necessary to verify the reproducibility of these results by increasing the sample size or using different populations.

Another limitation of this study is that it is based on DNA methylation data obtained from blood samples, specifically, PBMCs and monocytes. Although most of the EWAS studies listed in the EWAS Atlas used blood samples, DNA methylation patterns can be different between blood cell types. To reduce the possible effects of different cell types, the CpGs whose longitudinal DNA methylation changes were associated with cell types were excluded. However, only six major cell types were considered in this study, and we cannot account for subtypes of blood cells, which can change along with the onset of immune disorders [57]. Therefore, the individual results obtained from each PBMC and monocyte sample were not discussed.

In addition, it is currently not possible to plausibly explain the results of every EWAS trait considered in this study. To clarify the functional relationships between individual traits and the longitudinal dynamics of DNA methylation status, further studies based on data from individuals are required that focus on each trait. However, we suggest that the longitudinal dynamics of DNA methylation are associated with specific biological functions, and thus, are an important clinical indicator. The longitudinal dynamics of within-individual and between-individuals DNA methylation of each CpG calculated in this study are publicly available in the iMETHYL database. We believe that this will provide a new perspective on the interpretations of published and future EWAS findings.

## Supplementary Information

**Additional file 1: Figures**. S1–S13.

**Additional file 2: Tables**. S1–S7. **Table S1**. Characteristics of the two study participants. **Table S2**. Numbers of CpGs of each category in each sample. **Table S3**. Enriched gene-ontology terms (biological process) among genes with stable or dynamic CpGs. Enrichment analyses were performed for each of the four samples. FDR-corrected *p*-values are presented. NE: not enriched. **Table S4**. Enriched gene-ontology terms (cellular component) among genes with stable or dynamic CpGs. Enrichment analyses were performed for each of the four samples. FDR-corrected *p*-values are presented. NE: not enriched. **Table S5**. Enriched gene-ontology terms (molecular function) among genes with stable or dynamic CpGs. Enrichment analyses were performed for each of the four samples. FDR-corrected *p*-values are presented. NE: not enriched. **Table S6**. Enriched Kyoto Encyclopedia of Genes and Genomes pathways among genes with stable or dynamic CpGs. Enrichment analyses were performed for each of the four samples. FDR-corrected *p*-values are presented. NE: not enriched. **Table S7**. Gene ontology-terms for *ISM1* and *GPR37*. Data obtained from QuickGO

## Data Availability

Summary statistics of longitudinal DNA methylation dynamics for each CpG are available in the iMETHYL database (http://imethyl.iwate-megabank.org). Individual-level data cannot be made publicly available to protect the privacy of the participants.

## Abbreviations

ANOVA: Analysis of variance
BMI: Body mass index
BMIQ: Beta-mixture quantile
BP: Biological process
CC: Cellular component
CD4^+^ T: Cluster of differentiation 4-positive T lymphocyte
CD8^+^ T: Cluster of differentiation 8-positive T lymphocyte
CDMV: Common DNA methylation variation
CpG: Cytosine phosphate guanine
EPIC: Illumina MethylationEPIC
eQTM: Expression quantitative trait methylation
EWAS: Epigenome-wide association study
FDR: False discovery rate
GO: Gene ontology
GPR37: G protein-coupled receptor 37
HM450k: Illumina HumanMethylation450 BeadChip
ISM1: Isthmin 1
KEGG: Kyoto encyclopedia of genes and genomes
MAF: Minor allele frequency
MF: Molecular function
OR: Odds ratio
PBMC: Peripheral blood mononuclear cell
PCA: Principal component analysis
PM 2.5: Particulate matter (2.5 µm or less in size)
RI: Reference interval
TSS: Transcription start site
UTR: Untranslated region
WGBS: Whole-genome bisulfite sequencing
