# Supplementary material for: Longitudinal DNA methylation dynamics as a practical indicator in clinical epigenetics
Source: Clin Epigenetics. 2021 Dec 13;13:219. doi: 10.1186/s13148-021-01202-6 (PMC8670275; doi:10.1186/s13148-021-01202-6)
Supplement: Supplementary file 1 — Additional file 1: Figures. S1–S13. [file 13148_2021_1202_MOESM1_ESM.docx]

Additional file 1

# Supplementary figures

**Fig. S1. Proportions of six major cell types estimated based on DNA methylation datasets.**

**Fig. S2. Conceptual figures demonstrating between- and within-individual reference intervals (RIs).** RI stands for the difference between the 95th and 5th percentile values of DNA methylation level (DNAm, %). Between-individual RI represents the diversity of DNAm across individuals, whereas within-individual RI represents the dynamics/stability of DNAm within an individual over time.

**Fig. S3. Distributions of within-individual RI and mean DNA methylation across 24 time-points in each individual/sample-type.** RI: reference interval.

**Fig. S4.** **Pairwise overlaps of CpG between each CpG category.** The number and color of each tile indicate the ratio of the number of CpGs in the X-axis that overlap with the CpGs in the Y-axis.

**Fig. S5.** **Pairwise overlaps of CpG annotation between each CpG category.** The number and color of each tile indicate the ratio of the number of regions of a certain CpG annotation category in the X-axis that overlap with those in the Y-axis.

**Fig. S6.** **Top 20 enriched gene ontology (GO) biological process terms in each sample and category.** The complete list of enriched terms is available in Table S1.

**Fig. S7.** **Top 20 enriched gene ontology (GO) cellular component terms in each sample and category.** The complete list of enriched terms is available in Table S2.

**Fig. S8.** **Top 20 enriched gene ontology (GO) molecular function terms in each sample and category.** The complete list of enriched terms is available in Table S3.

**Fig. S9.** **Top 20 enriched KEGG pathways in each sample and category.** The complete list of enriched pathways is available in Table S4.

**Fig. S10.** **Pairwise overlaps of enriched term and pathway between each CpG category.** The number and color of each tile indicate the ratio of the number of enriched terms/pathways in the X-axis that overlap with those in the Y-axis. There were no enriched terms/pathways in several dynamic CpG categories (indicated with gray).

**Fig. S11.** **Characteristics of microarray probes of each CpG category.** (a) Proportions of CpGs measured by Infinium I and II types. (b) GC contents of probes for CpGs in each category. Asterisks indicate the significant difference of GC contents compared to the HM450k background. The Wilcoxon signed rank test was performed, and the Bonferroni-corrected significant threshold was used (0.05/12 comparisons)**.** (c) Overlaps of enriched terms/pathways between Infinium I and II CpGs. The number of enriched terms/pathways are presented. Only stable CpGs were considered in the analyses. Procedures of enrichment analyses were the same as those described in the main text.

**Fig. S12.** **EWAS marker likelihood for CpGs in each CpG category.** Categories with a positive odds ratio that significantly deviated from 1 are denoted with asterisks.

**Fig. S13.** **Trait-specific EWAS marker likelihood for CpGs in each category with 95% confidence interval.** Categories with positive odds ratios that significantly deviated from 1 were denoted by asterisks.
